# MyoLoop: Redefining cardiac research with advanced tissue simulation

**DOI:** 10.1113/EP091577

**Published:** 2024-01-18

**Authors:** Matthew Reily‐Bell, Rajesh Katare

**Affiliations:** ^1^ Department of Physiology HeartOtago University of Otago Dunedin New Zealand

**Keywords:** cardiac electrophysiology, cardiac remodelling, myocardial slice, MyoLoop

1

A key challenge in the translation of cardiovascular disease therapies to the clinic is the lack of models that closely replicate the complexity of the human heart. Although large animal models resemble the human heart, they are impractical owing to high costs and time requirements. Although more affordable two‐ and three‐dimensional cell culture models are being developed, they often lack key properties, such as metabolism, contractility and electrophysiology (Perbellini & Thum, 2020).

In 2019, the advent of electromechanical cultivation chambers enabled the long‐term cultivation of living myocardial slices (LMS) (Perbellini & Thum, 2020; Watson et al., 2019). These slices, 100–400 μm in thickness, maintain the native cellular composition, organization and interactions of cardiac tissue. A key innovation in these chambers, developed by Watson et al. (2019), was their ability to apply mechanical load to cells, thus preventing cardiac remodelling. Subsequent improvements have enhanced the ability of the system to replicate physiological mechanical load (Miller et al., 2022; Pitoulis et al., 2022). Pitoulis et al., 2022 developed a prototype cultivation chamber that uses a three‐element Windkessel (3EWK) model, allowing for the simulation of work loops that closely mimic the in vivo cardiac cycle phases of isometric contraction, ejection, relaxation and diastolic filling. However, two major challenges persisted: the complexity of the system and its low throughput, limited to culturing a single LMS at a time.

In this issue of *Experimental Physiology*, Pitoulis et al. (2024) introduce MyoLoop, a user‐friendly, benchtop LMS culture system that enhances accessibility and practicality in laboratory settings (Pitoulis et al., 2024). This system ingeniously incorporates the 3EWK model to simulate the physiological mechanical load of the heart in vivo. Detailed in the paper are the meticulous assembly of the MyoLoop and the sophisticated software development necessary for operationalizing the 3EWK model to generate work loops. A key aspect of MyoLoop is its near‐complete self‐sufficiency, needing only an external peristaltic pump for recirculation of medium. Its design also includes a detachable cell culture chamber, significantly simplifying the sterilization process. The user interface of MyoLoop is highlighted for its capability to record data either continuously or at set intervals, a feature crucial for optimizing data storage. Pitoulis et al. (2024) provide examples of simulating diverse mechanical loads using MyoLoop. To validate the efficacy of the system, LMS were subjected to three distinct preloads, transitioning from isometric modelling to the 3EWK mode, where dual afterloads were used to establish work loops. This 72 h culture demonstrated remarkable accuracy in LMS looping when operating in 3EWK mode, closely matching the anticipated waveforms. Moreover, the adaptability of MyoLoop in adjusting the LMS work loop based on the force exerted by the LMS underscores its advanced capability in simulating the evolution of contractile dysfunction, marking a significant leap in the field of cardiac research.

The development of MyoLoop, a largely self‐contained system with intuitive software, addresses the complexity issue identified by Miller et al. (2022) in the prototype of the system. Although its throughput capacity, currently accommodating two LMS, is still limited in comparison to Fisher's eight or Miller's twenty‐four system, it demonstrates the potential for managing multiple LMS simultaneously, indicating the possibility of higher‐throughput models in the future (Miller et al., 2022; Pitoulis et al., 2024).

In conclusion, the development of MyoLoop marks a significant advancement in cardiovascular research, addressing previous limitations/challenges in modelling heart tissue complexity and processing capabilities. Its innovative approach to mimicking contractile dysfunction offers broader applications, extending to studies of other organs, such as the stomach, thus reducing dependence on animal models. Looking ahead, the potential applications of this technology in personalized medicine are vast. The utility of MyoLoop in personalized medicine lies in its capacity to develop patient‐specific models. By simulating heart tissue dysfunction, it allows for the creation of individualized treatments based on the unique cardiac profile of an individual. This approach is especially promising in development of induced pluripotent stem cell‐engineered heart tissues, which can be tailored to match the specific genetic and cellular characteristics of a patient. This precision in treatment design enhances the effectiveness and safety of therapies, paving the way for more personalized and targeted health‐care solutions. However, realizing its full potential requires extensive future research. This includes rigorous clinical trials to ensure the safety and effectiveness of treatments derived from this technology. MyoLoop stands at the forefront of advancing more accurate and patient‐centric medical interventions, signalling a major leap forwards in health‐care innovation.

## AUTHOR CONTRIBUTIONS

Matthew Reily‐Bell conceived and wrote the first draft of the manuscript. Rajesh Katare conceived and provided extensive revision of the manuscript. Both authors contributed equally to the writing of the manuscript. The final version of the manuscript has been checked for accuracy and approved by both authors.

## CONFLICT OF INTEREST

The authors declare no conflict of interest.

## FUNDING INFORMATION

M.R.B. is supported by a University of Otago PhD scholarship.
